# Microfluidic Formulation for Biomedical Applications

**DOI:** 10.3390/ph16111587

**Published:** 2023-11-09

**Authors:** Kieu The Loan Trinh

**Affiliations:** BioNano Applications Research Center, Gachon University, 1342 Seongnam-daero, Sujeong-gu, Seongnam-si 13120, Republic of Korea; tktloan@gmail.com

## 1. Introduction

Microfluidic technology was recognized in the 1980s when the first micropumps and micro-valves were developed to manipulate fluids for biological applications [1]. Microfluidics can be described as a science in which researchers study and exploit the behavior of fluids confined to small volumes, ranging from nano- to microlitter, through the device’s channels with diameters in a micrometer scale [2]. Given the small scales, the fluid patterns are a unique phenomenon, more controllable, advanced laminar flow, and fluids move effectively in smooth paths and layers. Typically, microfluidic devices are devised according to liquid propulsion types involving pressure-driven, capillary, acoustic, electrokinetic, and centrifugal systems in which the fluids can be controlled and manipulated [3]. The advanced features of microfluidic devices involve minimizing material and energy consumption, controllable reaction conditions, high-throughput signal, and precise sampling processes, enabling their widened applications in many fields, such as micro- and nano-fabrication, biological analysis, diagnostics, and drug development [4,5,6].

**Figure 1:**
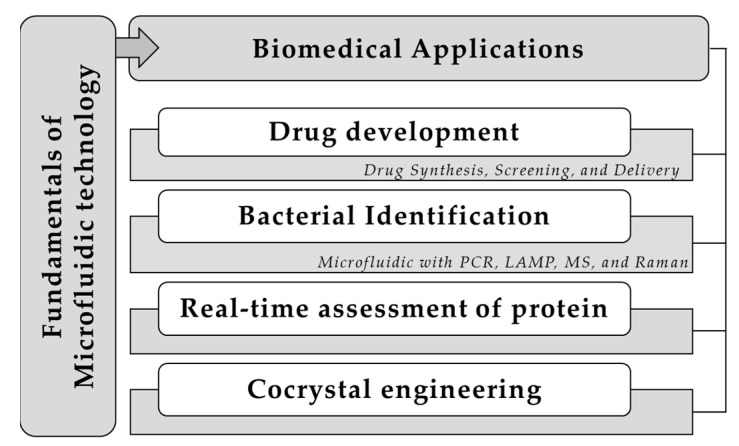
Microfluidic formulation for biomedical applications involving drug development, bacterial identification, real-time assessment of protein, and cocrystal engineering.

Along with microfabrication technology developments that feature microstructure fabrication, parts assembly, and microchannel surface modification, microfluidic technology flourished from its unique benefits such as reduced reagent volume, automated and multiplexed reactions, and miniaturized system. As a result, microfluidic technology was exceedingly incorporated into various biomedical applications to achieve precisions that cannot be realized in bulk processing. In light of the recent widespread and sudden interest in lipid nanoparticle-based drugs, which utilize microfluidic chips to achieve lipid-component assembly and drug/nucleic acid loading, microfluidic technology in pharmaceutical and biomedical applications is expected to grow larger. Moreover, microfluidic platforms can be adopted for fast toxicity screening by employing concentration gradient generators and cell culture chambers. It is also expected to play an important role in developing personalized medicine by isolating and culturing cells from patients, such as iPSC-derived cells or cancer cells, to screen for drug efficacy. In this connection, this Special Issue of *Pharmaceuticals*, “Microfluidic Formulation for Biomedical Applications”, aims to provide a foundation and advancement of microfluidic technology in the biomedical field. The original research and comprehensive review papers in this Special Issue have explored and updated the new progress designs and applied microfluidics in drug development, bacterial identification, real-time assessment of protein, and cocrystal engineering (Figure 1).

## 2. Droplet-Based Microfluidic for Pharmaceutical Applications

The droplet-based microfluidic platform involves strategies for the formation, manipulation, and control of uniform and micron-size droplets that are isolated using an immiscible carrier fluid [7]. This platform has shown great potential for pharmaceutical applications, especially in drug development, as a dynamic branch of microfluidic technology. Drug development implementation involves fundamental synthesis strategies, screening, delivery, and evaluation [8]. In this Special Issue, the article by Trinh et al. (contribution 1) has provided a comprehensive summarization of the recent progress of droplet-based microfluidics for biomedical applications. Specifically, the fundamentals of droplet-based microfluidics were described, including droplet-based microfluidics device fabrication, droplet generation and manipulation, and open droplet microsystem. The authors also highlighted and discussed the design and use of droplet-based microfluidic for drug synthesis, screening, and delivery in depth. These droplet systems have been introduced as microreactors to produce solid particles, which are more challenging if synthesized through conventional methods such as indigo synthesis. The droplet systems have advanced drug screening involving natural antibiotics, bacteria, and extracellular products as pharmaceuticals. Moreover, drug delivery is a crucial strategy and aspect of drug development. Utilizing droplet-based microfluidic facilitates the control of the delivery and release of appropriate concentrations of drugs in the body. Moreover, the implementation strategy of droplet-based microfluidic technology remains some challenges that should be improved involving (1) the fabrication techniques of the device, (2) the stability of the droplet, (3) fluorescent market selection, and (4) the compatibility of the drug molecules, oil, surfactant, as well as materials of the devices.

## 3. Bacterial Identification

Pathogen detection and analysis using microfluidics have exhibited benefits such as cost-effectiveness, time-saving traits, accuracy, high sensitivity, and capacity to develop portable and smart point-of-care devices [9,10]. Due to their flexibility and small size, microfluidic devices can be integrated with different sensitive analytical systems as multifunctional platforms. This Special Issue includes a systemic summarization of using microfluidics to identify and analyze bacteria by Daniel et al. (contribution 2). In this paper, the microfluidics devices using polymerase chain reaction (PCR), loop-mediated isothermal amplification (LAMP), matrix-assisted laser deposition/ionization mass spectroscopy (MALDI-ToF MS), and Raman spectroscopy for bacterial identification have been thoroughly discussed and compared. In general, combining these techniques allows the detection and analysis of the bacteria without requiring bacterial cultures. The advantages and disadvantages of these technologies have been discussed in detail.

## 4. Real-Time Assessment of Protein

The liquid-handling capacity and fewer sample requirement of microfluidics, with precise manipulation of loading samples, enables their high potential application in biomolecule analysis such as nucleic acid, protein, and hormone [11,12]. Microfluidic technology has provided bioanalytical systems with low cost, high accuracy, high resolution, and simple operation [13]. Kuzman et al. (contribution 3) have recently proposed using microfluidic technology for the real-time analysis of protein particles. In this study, the microfluidic device was created using the standard soft-lithography method with a chamber (microcavity) length of 250 µm, width of 100 µm, and depth of 40 µm. Protein particles could transfer into the chamber using optical tweezers. The effective flow-free microfluidic chamber enables particles to remain undisturbed and be analyzed in real time. The as-prepared microfluidic system allowed for the characterization of morphology, size, and intermolecular forces of the protein under changing of buffer and urea concentration. The biophysical properties of protein particles and mono-protein also was performed using the microfluidic chip.

## 5. Cocrystal Engineering

Cocrystallization has been considered an effective method to alter the physicochemical properties of active ingredients in pharmaceutical drugs. The cocrystals can be created through various processes, including grinding (neat or dry and solvent-assisted grinding), slurry conversion, antisolvent addition, and solvent evaporation [14,15]. Obtaining good structure and quality of cocrystal particles required developing new or/and a combination of various fabrication methods. Microfluidic technology offers great potential for cocrystal engineering, such as allowing accurate fluid loading and mixing ratio to enhance cocrystallization efficiency [16]. Recently, Kara et al. (contribution 4) reported using microfluidic devices developed with a stereolithography (SLA) 3D printer method to control cocrystals of sulfadimidine (SDM) and 4-aminosalicylic acid (4ASA). Tinkercad^®^ (Autodesk^®^, San Franciso, CA, USA) was used to design the microfluidic devices, which were then developed by Anycubic^®^ Photon Mono X SLA printer with a photocurable resin polymerized at 405 nm. The microfluidic devices with a length of 8.2 cm, width of 3.5 cm, and height of 0.7 cm were printed. These devices contain channels with a diameter of 1 mm and average surface roughness of 0.156 ± 0.106 µm were utilized to control the nucleation rate and cocrystal behaviors of SDM:4ASA. This work proposed for the first time coupling microfluidic devices with a fluidized bed for continuous manufacturing of cocrystallization. Interestingly, 0.1 PVP K25 was proved to avoid crystal formation in microfluidic devices during fabrication processes, resulting in more homogenous particles formed. The SDM:4ASA cocrystals were successfully achieved via integrated microfluidic devices with better morphology and distribution of formed sphere particles than the solvent evaporation method.

## 6. Conclusions

The findings of the papers presented in this Special Issue rapidly contribute to exploring and expanding new applications of microfluidic technology in biomedicine. We hope the published original research articles and comprehensive reviews will be attentive and appeal to a broad readership of biology, chemistry, pharmacology, and materials science.

## References

[1] Burklund A., Tadimety A., Nie Y., Hao N., Zhang J.X.J. (2020). Advances in Diagnostic Microfluidics. Adv. Clin. Chem..

[2] Limbut W., Promsuwan K., Kongkaew S., Thavarungkul P., Mak W.C. (2023). Emerging Functional Materials for Microfluidic Biosensors. Microfluidic Biosensors.

[3] Van Den Berg A., Craighead H., Yang P., Mark D., Haeberle S., Günter A., Roth G., Felix Von Stettenz A., Zengerlez R. (2010). Microfluidic Lab-on-a-Chip Platforms: Requirements, Characteristics and Applications. Chem. Soc. Rev..

[4] Zhang H., Yang J., Sun R., Han S., Yang Z., Teng L. (2023). Microfluidics for Nano-Drug Delivery Systems: From Fundamentals to Industrialization. Acta Pharm. Sin. B.

[5] Zhao P., Wang J., Chen C., Wang J., Liu G., Nandakumar K., Li Y., Wang L. (2022). Microfluidic Applications in Drug Development: Fabrication of Drug Carriers and Drug Toxicity Screening. Micromachines.

[6] Niculescu A.G., Chircov C., Bîrcă A.C., Grumezescu A.M. (2021). Fabrication and Applications of Microfluidic Devices: A Review. Int. J. Mol. Sci..

[7] Moragues T., Arguijo D., Beneyton T., Modavi C., Simutis K., Abate A.R., Baret J.C., deMello A.J., Densmore D., Griffiths A.D. (2023). Droplet-Based Microfluidics. Nat. Rev. Methods Primers.

[8] Mak K.K., Pichika M.R. (2019). Artificial Intelligence in Drug Development: Present Status and Future Prospects. Drug Discov. Today.

[9] Lonchamps P.L., He Y., Wang K., Lu X. (2022). Detection of Pathogens in Foods Using Microfluidic “Lab-on-Chip”: A Mini Review. J. Agric. Food Res..

[10] Li W., Ma X., Yong Y.C., Liu G., Yang Z. (2023). Review of Paper-Based Microfluidic Analytical Devices for in-Field Testing of Pathogens. Anal. Chim. Acta.

[11] Berlanda S.F., Breitfeld M., Dietsche C.L., Dittrich P.S. (2021). Recent Advances in Microfluidic Technology for Bioanalysis and Diagnostics. Anal. Chem..

[12] Xu X., Cai L., Liang S., Zhang Q., Lin S., Li M., Yang Q., Li C., Han Z., Yang C. (2023). Digital Microfluidics for Biological Analysis and Applications. Lab. Chip.

[13] Liu Q., Wei H., Du Y. (2023). Microfluidic Bioanalysis Based on Nanozymes. TrAC Trends Anal. Chem..

[14] Carneiro R.L., de Melo C.C., de Alvarenga B.R., Dayo Owoyemi B.C., Ellena J., da Silva C.C.P. (2022). Mechanochemical Synthesis and Characterization of a Novel AAs–Flucytosine Drug–Drug Cocrystal: A Versatile Model System for Green Approaches. J. Mol. Struct..

[15] Pawar N., Saha A., Nandan N., Parambil J.V. (2021). Solution Cocrystallization: A Scalable Approach for Cocrystal Production. Crystals.

[16] Puigmartí-Luis J. (2014). Microfluidic Platforms: A Mainstream Technology for the Preparation of Crystals. Chem. Soc. Rev..

